# The Condition of the Insane

**Published:** 1858-01-01

**Authors:** John Hawkes


### Art. IV.—THE CONDITION OF THE INSANE.

BY JOHN HAWKES, M.R.C.S., ETC.

If one particular feature of the epoch in which we live deserves to be characterized as a "sign of the times," there can be no hesitation in denoting the general attention and almost universal sympathy with which our insane are regarded as a striking circumstance in the history of this present age, directly pointing, as it does, to the influence of increasing Christian civilization in ameliorating the condition of all mankind, and of promoting among its different classes a kindly spirit of mutual regard, leading to the exercise of those heaven-born virtues through which man, not content with raising himself, stretches forth his hands to lift his fallen brother to the dignity of a lord of the creation.

We need not the perusal of reports to assure us how heavy must be the burden of insanity to a nation; but those who least regard the matter may well be appalled to hear that in England and Wales there are 21,311 insane persons under treatment in various asylums; there are some, perhaps, besides these, beyond the cognizance of Government inspectors, and, in addition, it may be feared many mental aliens are at large on society. It is therefore almost impossible to arrive at a positive appreciation of the amount of insanity existing under different forms throughout the country. It will suffice, however, for my purpose to consider this army of insane under two chief divisions—first, those that are met with in the hands of private individuals; and, secondly, such as are treated in the wards of public asylums. Of the former, we are informed by the last Report of the Commissioners in Lunacy, there are 2578 in metropolitan, and 2598 in provincial licensed houses. Out of one hundred and sixteen private asylums, not more than forty-six are entrusted to the hands of persons destitute of any professional qualification; these are doubtless humane and enlightened men and women, whose high aim it is to promote the restoration of reason to their unhappy patients, and to "comfort the feeble-minded," quite irrespective of any other considerations, though there may be some cause for apprehension that, amidst the anxieties attendant on so important a charge, precepts of trade are not wholly ignored, nor a little brisk business, strongly partaking of speculation, altogether disregarded; that even under luxurious refinement lurks gilded misery, and in the chambers of first-class patients the air of a commercial enterprise;—else why these advertisements setting forth in glowing eulogy the sweet Elysium, or why the trumpet-tongued announcement bidding patients walk up hither? It is scarcely affirming too much to say that the greater number of such establishments are little short of private hotels, and that the benevolent proprietor or landlord sees no harm in mixing up philanthropy and trade; indeed, if he can lay claim to a slight degree of taste, it is even possible his house may possess external features, at least, rather pleasing, provided the illusion is not too abruptly dispelled by some enormity in the decorative department; for there can be nothing more painful to look upon than one of these houses "licensed for the reception of the insane," plastered and painted in the highest style of embellishment, and surrounded by gaudy flowers, while through the narrow, small-paned windows come groans and sighs, or curses long and loud; pale, speechless faces peering through iron bars, and restless eyes, which once shone with love and gladness, gleaming forth in frenzied woe. Equally sad, perhaps more so, to witness a place of detention for the mental afflicted, in that state of dilapidation which says so plain 'tis not worth laying out a sixpence on the property, for the sombre, damp-stained walls are half-concealed by straggling, ill-grown trees, and all around an air of desolation. The "large and extensive pleasure grounds," overrun with nettles and long rank grass, and the paths, untrodden by human feet, are green with moss and slime. Could we get round to the back of the premises, more than one window would be seen boarded up to within a foot of the top, while a few remaining fragments of glass testify to the raving madness of some former occupant of that dreary apartment. And though, for the sake of humanity, we may hope such establishments are few and far between, it is well to remember that till within the present half century this was by no means the case; and much as we have advanced in the treatment of our insane, both in public and private asylums, there yet remains a great deal to be accomplished before we can be beyond reproach. Let us never forget the debt we owe to the early champions of reform. Under their gallant leadership, the onward current of public opinion has swept away many foul and cruel abominations, which full long held their sway; but the name of Pinel will remain to future ages emblazoned, with undying lustre, in his country's scroll of fame. Genius alone may command respect, scientific skill may claim admiration, but philanthropy that spends and is spent in the cause of humanity deserves our highest praise. It is, indeed, refreshing to turn for a moment from considering the wretched shifts and unprincipled expedients of worthless, low-minded men, who traffic in the harrowing misfortunes of madness, and drive bargains with friends hardly less to be pitied than the miserable objects of peculation—it is, I say, delightful to turn away from such as these to contemplate the pure, unselfish devotion of those influenced by higher and purer motives; but though the vast results of their humane exertions are readily obtained by the wealthy, and are even consecrated to charity, a large and important section of the community, known as the middle class, is placed in some degree beyond the direct influence of good which ever surrounds, as a halo of light, the labours of the true physician. It should be remembered, until of late years, how large a proportion of the licensed houses consisted of second-rate establishments—mere lodging-houses for the moral restraint of the insane. Their inmates are generally respectable tradesmen, or professional men of reduced means: occasionally, persons of considerable property are placed by considerate friends within their walls. The proprietor, if not himself a tradesman—either a retired victualler, gardener, or ambitious cobbler, as I have known to be the case—will probably be a member of the medical profession, who, from psychological zeal, broken health, or disinclination for more arduous professional pursuits, has applied himself to this calling. I cannot help thinking, if all proprietors were required to possess a medical education, it would be better for those consigned to their care. However this may be, it was seldom that men of great psychological talent, or otherwise highly gifted, were at the head of these establishments; indeed it is well when the medical officer, or superintendent, was not simply a puppet in the hands of an unscrupulous speculator, who really managed the asylum. From the consideration of these facts, a forcible argument can be deduced in favour of ameliorating the condition of middle-class patients, by placing them, at moderate charges, under the care of able men, qualified by education and experience, and chosen in virtue of their high attainments, for the discharge of this high function. The best method of effecting this object will next engage our attention. An establishment calculated for the reception of insane persons from the middle ranks of society, formed on a similar plan to that of a proprietary college, with working regulations, somewhat in the manner of our best public asylums; the whole invested in the hands of shareholders, or subscribers, who would govern by a committee of directors, having, as above stated, a resident medical superintendent, with additional officers as in other institutions. Here is a step from the narrow despotism of an ignorant or unprincipled proprietor to the comparative freedom and wholesome management of a little republic. One individual mind would no longer be permitted to cramp the healthy working of the whole, nor to fetter, with jealous regulations, the energetic will of an earnest and devoted officer; but all may "work together for good," inspired by unanimous principles, and having one end in view. Indeed, I am of opinion that the plan here advocated, viz., a large proprietary institution, containing three or four hundred souls—not more, perhaps less—is the best, under certain conditions, that can be devised for the required purpose. In an asylum of this kind every guarantee must exist for the right treatment of its inmates: there can be no attempt at dishonesty, if the reports of its committees are duly submitted to public inspection; neither can inefficient medical aid be a hindrance or defect, if the managers take pains to select an officer of reputed acquirements in this department of his profession. And here I may take occasion to suggest an alteration in the titles affixed, in the present system of asylum government, to the principal officer of the institution. The term "Superintendent," though good enough in the days of leg-locks and keepers, seems hardly in accordance with the duties of one whose special province it is to

> "Fetter strong madness in a silken thread—
> Charm ache with air, and agony with words;"

and I have reason to believe it is indirectly repudiated by some of the most enlightened physicians in charge of public asylums. "Principal," or "Senior Medical Officer," is more in keeping with the views of the present day. The latter implies that another medical officer is resident in the house. With that liberality of sentiment characteristic of a learned profession, the second, or junior officer, may aptly be termed "Vice-Principal," or "House Surgeon." In very few asylums, the questionable title of "Medical Assistant" is assigned him, as though under any other name he would approach too near the dignity of the chief in command. The more general title of "Assistant Medical Officer" is, however, less objectionable. Some exception has been taken, I believe, to the name of "Matron," but it is difficult to find a better; perhaps that of "Mistress," or in some cases "Lady Superintendent," may be considered preferable: after all, it matters little—

> "A rose by any name would smell as sweet."

Lastly, the appellation of "Steward" may advantageously be exchanged for "Manciple," as strictly in keeping with ancient custom, and equally applicable to the duties of his office. I shall not venture further to sketch the outlines of a system such as now suggested, but will rather quote a striking and interesting account of an asylum, somewhat of this kind, in Flanders, taken from a review by the talented pen of Dr. Lockhart Robinson, in a late number of the "Asylum Journal:"—

"It is an old and picturesque building, and part of it being castellated and constructed on a bridge which crosses the canal, it has the appearance of an ancient fortress. One side rises from a green turf bank on the water's edge, and the old dark, russet wall is partly covered with ivy, from which peep out the battlements crowning it. The gate is studded with nails, but scarcely in consequence of the present use of the house, as kind and gentle usage of the mental afflicted has here entirely superseded force and restraint. Nevertheless, there are upwards of three hundred *aliencés* in this house, under the superintendence of forty sisters. The patients are of three classes or grades in society. The most numerous is, of course, that of the *indigens;* next in number is that of the *bourgeoisie*, or middle class, who pay a moderate sum, and enjoy the advantage of semi-private apartments; and lastly, that of persons of family and fortune, who can, if they please, be accommodated with *salons*, *chambres à couches*, and *cabinets de toilette*, as elegant as anything to which they have been accustomed in their own luxurious abodes. Besides these divisions, there are special wards, padded rooms, and private gardens for those whose condition renders them dangerous and undesirable companions for the rest. The house is very extensive, and we were occupied a long time in merely walking over it. Above the cloister, which, as it were, lines the quadrangle, is an outer gallery, very prettily trellised and intertwined with creepers, serving both for ornament and security. There is an *aumônier*, and mass is said daily in the chapel, to which the inmates are allowed access at all times. . . . . Of the lower class of patients, those who are sufficiently sane are employed in various ways in the *ménage*. A large number were employed in washing in the laundry. . . . . Of the second class, about forty were manufacturing lace, and appeared perfectly rational; this lace is all sold for their own benefit, and the proceeds serve to supply them with such little *douceurs* as the charity cannot afford them. In another room, some were making clothes for their own wear, while parties of others were amusing themselves with cards or dominoes. Of the upper class, many remain in their own apartments, either from choice or because they are not fit to leave them; but about half-a-dozen were seated in an arbour formed in their own private garden, which is very tastefully laid out. One or two were engaged in fancy work, two were conversing apparently very rationally, and another was reading. A sister was with them. The Rev. Mother told me these were all *personnes de considération*. There is a common dining-room for these patients, where all who are not confined to their own apartments meet for meals, unless, as in some cases, they prefer solitude."*

* "Flemish Interiors." Longman. 1856.

However peculiar this picture may appear to our English notions, there are many points therein shown which deserve our imitation. I will say nothing of the laudable devotion of these Sisters of Mercy. Our own country has evinced her ability to send forth women no less distinguished for the highest attributes of their sex, as for every Christian virtue; and we may be sure if ever a cry should be raised from the dwellings of those afflicted with the direst of human calamities—if it shall be proclaimed that the nursing hand of woman must distinguish the treatment of the insane—then we shall witness a spectacle such as described in the passages above quoted, where religion, self-devotion, and heroism of no common type, combine to render the weaker sex ministering angels of God. It must have occurred to those who entertain feelings of pity and sympathy for their poorer neighbours, that many, from inability to maintain a relative as they could wish under private treatment, are constrained to consign that beloved one, whether parent, husband, or child, to the wards of the county asylum, where he is left to struggle through the fell disease, associating with others below him in social standing, and who are little disposed to make allowance—if, poor souls, they could—for his sensitive or sorrowing mind. I have known some instances of this, where the affliction must cut, if possible, with a double edge—wounding the self-respect of the patient, who feels his degradation; and galling to the proper pride of his relatives, who for the first time become dependents on public charity. To meet this evil, I particularly uphold the system of proprietary asylums, as one under which those who are much above pauperism, and yet, from some circumstances, are unable to bear the expense of a good private "retreat," may be admitted to the advantages of skilful medical aid, with liberal and humane treatment, without the pain of losing caste, or suffering the additional pang which a pauper's dress may entail. This will appear at first rather a difficult problem; let us see how it may best be solved. The simplest plan for affording accommodation to non-paying residents in any community, must evidently be by placing the burden of their keep on the shoulders of the rest; and, in an establishment of the kind we are now considering, this could be easily managed by common foresight and judgment, in so arranging the scale of charges that a certain number, say five per cent., should receive themselves the gratuitous benefit of the institution. In course of time, we may reasonably expect charitable persons, governors and others, would bequeath sums of money for the purpose of creating, by endowment, additional freeholds, as they may be termed, to be filled by deserving cases, answering very much to scholarships in our universities, by aid of which many a poor scholar has become a great man, and lived to bless the founder.

What an opportunity for the noble employment of wealth! what ripe occasion for putting one's hand and seal to a life of good works, would such a scheme afford!—opportunity and occasion that many a dying man would grasp with joy, over whose tomb should be shed the grateful tears of the afflicted and destitute, to whom he had shown himself a brother and a friend. The middle class, when overtaken by insanity, is, as a rule, more to be pitied than the poor; for the professional man must then exchange the comforts of his home, and the kind attention of friends, for the barren consolation of strangers and the discipline of a madhouse. Even where no real cruelty exists, there is too often the painful apprehension present, and the feelings of enforced subserviency to the *dictum* of a master whose humanity is suspected or questioned. On the other hand, the labouring man is removed from his squalid home of sorrow to a clean, well-kept asylum; trained attendants, carefully watched, wait upon him; full meals of well-cooked food are supplied him, and some degree of cheerfulness reigns around. While mixing with the rest, he has opportunities for forgetting, in part, his trouble. The social and domestic habits of this latter class are also very much in his favour. Accustomed to render obedience to the authority of their employers, the discipline of asylum government, rigorous though it may be, is no fresh trial; and, moreover, habituated as the poor are to holding their griefs in common, it becomes a less difficult matter to find some among his new comrades who will listen to his tale of tears, than in the case of the better educated, more elevated member of society, who, from the common exclusive habits of our Saxon race, is little wont to make others companions to his distress, or to pour his complaints into the ears of strangers. Thus the latter is more isolated, and less disposed to forget his melancholy by mixing in the crowd; while from his position, hard labour, as husbandry and other useful occupations, which can be rendered to a certain degree compulsory in a pauper asylum, must be mainly left to his natural taste or disposition. Therefore the position of the insane pauper, supposing him to rank rightly in the lower or labouring class, is in some respects essentially superior to that of the more educated man, especially if the latter has not the means of diverting his mind by those pastimes and indulgences which the affluent can alone enjoy. Here we see the want of suitable accommodation for the mentally afflicted in the middle class of society—a want, the pressing requirements of which are such, that neither time should be lost nor expense spared in removing the evil and reproach. It is time indeed that the middle classes should protect themselves from evils which by sufferance must increase. It is vain to wait while all around are bestirring themselves and girding on their armour; while the luxurious tendencies of the wealthy are inducing their own sure results, the natural appetites of the lower classes are becoming daily keener and less readily satisfied. Their intellects, sharpened by want, and a high-pressure system of education such as our fathers never dreamed of, must sooner or later bring them in collision with those immediately above them. But already the warning note has sounded; the slow upheaving of society has excited general attention; and a safety-valve has been opened, whereby the dangerous pressure on the condensed middle class will be for a while obviated or lessened. We now hear everywhere of "middle class education;" the universities, those elderly maiden sisters who have so long guarded the keys of knowledge, begin to take alarm. Awaking, they ask, What is this? What must we do? Vexatious and absurd regulations that have effectually barred the profession of arms from the sons of poor gentlemen, seem now about to be removed; while the opening out of our colonies, and the increased accommodation of the world, to speak metaphorically, must serve to relieve a dangerous plethora throughout all branches of the community. Another and still more forcible argument for the erection of proprietary asylums, in which the treatment of mental diseases shall be conducted on a system superior to any at present in vogue, will conclude this part of my subject. It is a fact, only too well known to those who are conversant with the practice of insanity, that many cases which, by early and judicious treatment, might have been restored to health, have, through neglect, or the untimely employment of remedies, degenerated into chronic mania and imbecility. This truth, which cannot be too widely disseminated, has again and again been brought forward to show the penny-wise and pound-foolish policy of poor-law guardians, who too frequently neglect placing a patient in the county asylum till his malady has become confirmed, while by heed to the early symptoms, and with due treatment, the individual might have been saved to his country and friends. But if instances of this woeful or wilful negligence are occasionally brought forward at the committees of public asylums, who can tell how many hundreds or thousands of private csses have, for want of timely recognition and the adoption of prompt measures, been irrecoverably lost? The desolate and empty mind, like some ruined castle abandoned to decay, that once by the employment of common skill might have saved from destruction, is now, alas! too far gone. Never can architect's hand replace that gilded capital; the ornate hall may never smile again. Who has not seen, and almost wept to view, that marvellous piece of creation, the human eye, marred by disease? The delicate and transparent cornea, through which has poured in by-gone days visions of light and beauty, now clouded and dull; the inner chamber a wreck, like the *boudoir* of a palace with its mirrors broken, its tapestry torn, its costly graces dimmed; and this sad havoc from no sudden, fierce invasion of disease, nor the fatal injury of a moment, but slow, continued, prolonged, and perhaps *untreated because unrecognised.* Such has been the progress of events which have ended in total ruin. And thus with the mind: that wondrous web of fine-spun fibre which we term the brain, has received, unknown, and hence unheeded, a fatal strain; or, in the natural progress of disease throughout the system, it becomes the seat of mischief, though the nature of this change, at once subtle and mysterious, is not, I may almost say cannot, be known; but now events occur which, to the educated eye, bespeak the presence of disease—just as the foul tongue or accelerated pulse tell of a vitiated or feverish body, so the wandering eye, the failing memory, the muffled speech, tell of causes at work which may end only in death. But what if, by early treatment, the dread result be changed to life and health? What if disease be driven from its citadel, and forced to evacuate its hold? What, in short, if man has it in his power to turn nature backward, and to keep, for a season, death itself at bay? Is this nothing? Is it not rather everything? and should we not hasten to avail ourselves of the advantage which a timely interference will give us for quelling, perhaps for ever, the direst of human disorders? It is only by the early treatment of insanity that we can reasonably hope to effect a lasting cure: vain is our attempt to pull down what fell disease has reared, should we come late to the task. Here, as everywhere, we must begin in time. It is not the fault of medicine if the public, from mistaken motives of economy, or a desire to keep well in spite of their malady, refuse to seek medical relief till the hour for active treatment has passed. Too often the hapless patient lies prostrate under the shadow of the grim king, the physician meanwhile seeking in vain for aught but a Euthanasia. In diseases, so called, of the mind, this most pernicious delay often results from a wish to keep the poor sufferer at home, under the impression that if removed to an asylum his recovery may be indefinitely postponed; or from a conscious inability on the part of his friends to place him in skilful hands. Here the talent and resources of a first-class institution, with the moderate charge of an ordinary second-rate one, would afford no excuse, and probably none would be wanted, for delaying to begin the cure. Much more may be added to this argument to prove the moral obligations under which we all live, not only to assist, but also to provide for and against common disasters and affliction to which, as one family, we are liable; let it suffice, however, that we bear in mind the consideration of our duties as neighbours, one to the other, and remember that in providing for the safety and well-being of others we provide for our own. A change is urgently required, and the sooner it is brought about the better for us all.

---

### Art. V.—PATHOLOGY OF INSANITY.

